# Drug-Induced Liver Injury Caused by Abiraterone Acetate in Patients With Prostate Cancer

**DOI:** 10.7759/cureus.83494

**Published:** 2025-05-05

**Authors:** Yoshihiro Nakagami, Toshiki Mugita, Masahiro Kurokawa, Sho Okada, Tatsuki Inoue, Kazuhiko Oshinomi, Masakazu Nagata, Takashi Fukagai

**Affiliations:** 1 Department of Urology, Showa University, Tokyo, JPN

**Keywords:** abiraterone acetate, drug-induced liver injury, metastatic castration-resistant prostate cancer, metastatic hormone-sensitive prostate cancer, r ratio

## Abstract

Background: Abiraterone acetate is indicated for high-risk metastatic hormone-sensitive prostate cancer (mHSPC) and metastatic castration-resistant prostate cancer (mCRPC). However, abiraterone acetate is significantly associated with drug-induced liver injury (DILI). In this study, we report severe cases of abiraterone acetate-induced liver injury in patients with prostate cancer.

Methods: This retrospective study uses medical records from September 2014 to November 2023. During this period, abiraterone acetate was administered to 126 patients at the Showa University Hospital in Japan. Of these, 113 patients were included in this study for analysis of the incidence of DILI. The incidence, timing, grade, and treatment of abiraterone-induced liver injury were investigated.

Results: The median age of the included patients was 76 years. There were 32 cases of upfront therapy, 63 cases of pre-docetaxel therapy, and 18 cases of post-docetaxel therapy. Seven patients had ≥ grade 3 DILIs and comprised three cases of upfront therapy, three of pre-docetaxel therapy, and one of post-docetaxel therapy. There were four cases of the hepatocellular type and three cases of the cholestatic type of DILI. Liver injury developed in all cases from the fourth week to the eighth week since drug administration. Abiraterone acetate was immediately discontinued in all patients with liver injury. The incidence of DILI in the upfront therapy group of this study was significantly higher. Liver metastases (OR = 6.667, p = 0.043, 95% CI (1.063-41.773)) and gamma-glutamyl transpeptidase levels (OR = 7.556, p = 0.013, 95% CI (1.534-37.214)) were significantly associated with grade ≥ 3 DILI in a single logistic regression analysis.

Conclusion: Severe DILI is a potential complication of the administration of abiraterone acetate. We recommend close monitoring for liver injury during abiraterone acetate therapy, especially in the first few months.

## Introduction

In 2020, prostate cancer was the most prevalent cancer affecting males in Japan [1]. The prognosis is typically poor in cases with distant metastases, such as to the bone, lung, and lymph, at diagnosis [2,3]. Prostate cancer is treated through surgery, radiation therapy, and drug therapy, which includes chemotherapy with docetaxel or cabazitaxel and hormone therapy with luteinizing hormone-releasing hormone and/or androgen receptor signaling inhibitor (ARSI). The ARSI treatment is currently considered the gold standard for hormone therapy. Abiraterone acetate, one of the ARSIs, is indicated for the treatment of high-risk metastatic hormone-sensitive prostate cancer (mHSPC) [4] and metastatic castration-resistant prostate cancer (mCRPC) [5,6]. Abiraterone acetate is significantly associated with drug-induced liver injury (DILI). However, few studies have summarized liver dysfunction in Japanese patients. Herein, we report severe abiraterone acetate-induced liver injury in patients receiving abiraterone acetate for high-risk mHSPC and mCRPC at our institution. This study aimed to determine the incidence, characteristics, and risk factors of abiraterone-induced liver injury in Japanese prostate cancer patients.

## Materials and methods

Sample collection

This study investigated the incidence, timing, grade, and treatment of abiraterone-induced liver injury. The study was conducted per the principles of the Declaration of Helsinki. It was approved by the ethical committee on research involving human subjects by the Institutional Review Board of Showa University (approval no. 2023-180-B) on November 17, 2023. 

The inclusion criteria were patients who received abiraterone acetate with high-risk mHSPC, pre-docetaxel mCRPC, and post-docetaxel mCRPC at Showa University Hospital (Tokyo, JPN) from September 2014 to November 2023. Of the total 126 patients, 113 were included in the study after excluding clinical trial cases and patients transferred from other hospitals. This study was a retrospective study using medical records. Abiraterone acetate was administered with prednisone 5 mg/day for patients with high-risk mHSPC and prednisone 10 mg/day for patients with mCRPC. The incidence of DILI was also analyzed using the Common Terminology Criteria for Adverse Events (CTCAE) version 5. Liver dysfunction was examined based on blood samples collected approximately every two weeks until 12 weeks after starting abiraterone acetate. Prostate-specific antigen (PSA), hemoglobin (Hb), WBC, platelet, albumin (Alb), aspartate aminotransferase (AST), alanine aminotransferase (ALT), total bilirubin (T-Bil), gamma-glutamyl transpeptidase (γ-GTP), alkaline phosphatase (ALP), and lactate dehydrogenase (LDH) were examined as blood tests. It should be noted that, in Japan, ALP was calculated using the Japan Society of Clinical Chemistry (JSCC) method until March 2020, and thereafter it was calculated using the International Federation of Clinical Chemistry (IFCC) method. In the current study, conversion was performed using the formula: ALP (IFCC) ＝ 0.35 × ALP (JFCC), which is provided by JSCC. The DILI can be categorized as hepatocellular, cholestatic, or mixed type based on the R ratio derived from the ratio of ALT to ALP [7]. 

Statistical methods

All analyses were performed using the STATA version 18 software package (Stata Corp. LLC, College Station, TX, USA). Statistical analysis was performed using single logistic regression analysis. The missing values were imputed using Markov chain Monte Carlo in the multiple imputation methods. The analysis was performed using 50 sets. Twenty-two missing values were identified in this study.

## Results

The pre-treatment characteristics of the patients are shown in Table 1. The median age was 76 years. Pre-docetaxel was the most common therapeutic line. Eight patients had liver metastasis. Although there were 125 abnormal values of all DILI, there were only 15 abnormal values of ≥ grade 3 DILI (Table 2). As the grade increased, the incidence of DILI decreased. The T-Bil abnormalities were most common, but grade 4 was not recognized. Seven patients had ≥ grade 3 DILI (Table 3). The AST and ALT levels were often elevated simultaneously in patients with ≥ grade 3 DILI. All cases of ≥ grade 3 DILI occurred from the fourth to the eighth week of abiraterone acetate treatment. Abiraterone acetate was immediately discontinued in all patients with ≥ grade 3 DILI. Taurine and ursodeoxycholic acid (UDCA) were used in one case, and UDCA was used in another case among the seven patients with ≥ grade 3 DILI.

**Table 1:** Characteristics of all patients using abiraterone acetate Hb: Hemoglobin; Alb: Albumin; AST: Aspartate aminotransferase; ALT: Alanine aminotransferase; T-Bil: Total bilirubin; γ-GTP: Gamma-glutamyl transpeptidase; ALP: Alkaline phosphatase; LDH: Lactate dehydrogenase

Characteristics	All patients (total n = 113), n (%)
Age, years; median (range)	75.44 (44–92)
PSA, ng/mL; median (range)	1351.34 (0.02–124692)
Treatment line, n (%)	
Upfront therapy	32 (28%)
Pre-docetaxel therapy	63 (56%)
Post-docetaxel therapy	18 (16%)
Metastatic site, n (%)	
Bone	94 (83%)
Lung	22 (19%)
Liver	8 (7%)
Lymph node	53 (47%)
Laboratory data	
Hb, g/dL; median (range)	12.08 (6.8–15.7)
WBC, /μL; median (range)	6415.92 (3100–12400)
Platelet (×10^4^), /μL; median (range)	24.61 (1.6–53.9)
Alb, g/dL; median (range)	3.78 (0.4–4.8)
T-Bil, mg/dL; median (range)	0.70 (0.30–1.6)
AST, IU/L; median (range)	26.80 (11–132)
ALT, IU/L; median (range)	18.64 (3–81)
γ-GTP, U/L; median (range)	23 (8–302)
LDH (IFCC), IU/L; median (range)	280.86 (97–3495)
ALP (IFCC), IU/L; median (range)	199.50 (22.33–2114)

**Table 2:** All grades of DILI DILI: Drug-induced liver injury; AST: Aspartate aminotransferase; ALT: Alanine aminotransferase; T-Bil: Total bilirubin; γ-GTP: Gamma-glutamyl transpeptidase; ALP: Alkaline phosphatase; DILI: Drug-induced liver injury

Grade	T-Bil	AST	ALT	γ-GTP	ALP	Total
Grade 1	27	23	14	4	23	91
Grade 2	6	0	4	4	5	19
Grade 3	1	3	1	4	2	11
Grade 4	0	1	2	0	1	4
Total	34	27	21	12	31	

**Table 3:** All cases of ≥ grade3 DILI DILI: Drug-induced liver injury; AST: Aspartate aminotransferase; ALT: Alanine aminotransferase; T-Bil: Total bilirubin; γ-GTP: Gamma-glutamyl transpeptidase; ALP: Alkaline phosphatase; R ratio: Ratio of ALT to ALP; G3: Grade 3; G4: Grade 4

Case No.	Therapeutic line	Age (years)	T-Bil (mg/dL)	AST (IU/L)	ALT (IU/L)	γ-GTP (U/L)	ALP (IU/L)	R Ratio	Type of DILI
Case 1	Upfront therapy	74	8.6 (G3)	35	79	611 (G3)	559	0.4	Cholestatic
Case 2	Upfront therapy	84	1.0	220 (G3)	87	120	211	1.1	Cholestatic
Case 3	Upfront therapy	87	1.1	1031 (G4)	1116 (G4)	26	109	27.5	Hepatocellular
Case 4	Pre-docetaxel therapy	67	1.3	265 (G3)	404 (G3)	110	136	8.0	Hepatocellular
Case 5	Pre-docetaxel therapy	75	1.1	128	52	670 (G3)	2163 (G3)	0.2	Cholestatic
Case 6	Pre-docetaxel therapy	81	1.1	49	126	366 (G3)	618	1.6	Cholestatic
Case 7	Post-docetaxel therapy	66	2.3	351 (G3)	1746 (G4)	542 (G3)	640	7.3	Hepatocellular

The DILI caused by abiraterone acetate improved relatively quickly by discontinuing the drug. Three patients were re-challenged with abiraterone acetate at the reduced dose, and one patient was re-challenged with the full dose. Thereafter, DILI did not recur in the re-challenged group. Among the remaining three patients with ≥ grade 3 DILI, two were treated with LHRH monotherapy, and one progressed to the best supportive care. The possibility of ≥ grade 3 DILI was examined using logistic regression analysis (Table 4). Liver metastases and γ-GTP were significantly associated with ≥ grade 3 DILI in single logistic regression analysis. No causal relationship was found between the occurrence of DILI and the timing of abiraterone acetate use.

**Table 4:** Logistic regression analysis for ≥ grade 3 DILI DILI: Drug-induced liver injury; Hb: Hemoglobin; Alb: Albumin; AST: Aspartate aminotransferase; ALT: Alanine aminotransferase; T-Bil: Total bilirubin; γ-GTP: Gamma-glutamyl transpeptidase; ALP: Alkaline phosphatase; LDH: Lactate dehydrogenase

Variables	Single logistic regression		
	OR	p-value	95% CI
Age (<75 years vs. ≥75 years)	0.983	0.983	0.210–4.614
Liver metastases	6.667	0.043	1.063–41.773
Treatment line (Upfront therapy vs. Pre-docetaxel therapy vs. Post-docetaxel therapy)	0.502	0.386	0.106–2.382
Hb (<14 vs. ≥14)	0.319	0.200	0.557–1.830
Alb (<4 vs. ≥4)	3.026	0.214	0.528–17.323
T-bil (<1.0 vs. ≥1.0)	0.594	0.740	0.273–12.919
AST (<30 vs. ≥30)	2.308	0.295	0.484–10.993
γ-GTP (<64 vs. ≥64)	7.556	0.013	1.534–37.214
LDH (JFCC:<222 vs. ≥222, IFCC:<245 vs. ≥245)	0.885	0.887	0.163–4.798
ALP (JFCC:<322 vs. ≥322, IFCC:<113 vs. ≥113)	1.017	0.983	0.217–4.769

## Discussion

In this study, we investigated the incidence of DILI in Japanese patients treated with abiraterone acetate. To the best of our knowledge, no studies have yet investigated DILI in Japanese patients in all periods, i.e., upfront therapy, pre-docetaxel therapy, and post-docetaxel therapy. Additionally, fulminant hepatitis and hepatic dysfunction may occur and lead to hepatic failure. Therefore, it is necessary to perform regular liver function tests during drug administration and closely monitor the patient's condition. 

In vivo, abiraterone acetate is rapidly hydrolyzed via the androgen synthesis pathway to abiraterone, which irreversibly and selectively inhibits 17α-hydroxylase/C17,20-lyase (CYP17) activity [8]. The CYP17 produces dehydroepiandrosterone and androstenedione, precursors of testosterone, from pregnenolone and progesterone. Thus, abiraterone inhibits CYP17 activity and testosterone and androstenedione synthesis. A previous study has shown that upon a single oral administration of 14C-abiraterone acetate to healthy adults, the abiraterone acetate rapidly hydrolyzes to abiraterone [8]. The abiraterone is predominantly metabolized in the liver, and 92% of the total plasma radioactivity was derived from a metabolite of abiraterone. Furthermore, 88% of the administered radioactivity was excreted in feces and 5% in the urine within 264 hours of administration. According to the Japanese package insert for abiraterone acetate, treatment should be discontinued for ALT and AST levels > 5 times the upper limit of normal levels or bilirubin levels > 3 times the upper limit of normal values. Once these tests normalize, a reduced dose of the drug should be carefully used for re-challenge. Notably, discontinuation becomes mandatory for cases with ALT and AST levels > 20 times the upper limit of normal or bilirubin levels > 10 times the upper limit of normal levels.

In our study, seven cases (6% of all cases) showed ≥ grade 3 DILI. Of these, three cases were in upfront therapy, three in pre-docetaxel therapy, and one in post-docetaxel therapy. The incidence of ≥ grade 3 DILI at each time was calculated. The result was that 9.3% were in upfront therapy, 4.8% in pre-docetaxel therapy, and 5.6% in post-docetaxel therapy. The incidence of liver injury varies across clinical trials. The COU-AA-301 trial showed an overall liver injury rate of 11%, with approximately 4% demonstrating ≥ grade 3 DILI [5]. Similarly, the COU-AA-302 trial documented an elevated ALT rate with 5% showing ≥ grade 3 DILI, and an AST elevation rate with 3% showing ≥ grade 3 DILI [6]. Conversely, the LATITUDE trial reported a low incidence of grade 1-2 (<1%) and grade 3 (<1%) liver injuries, with no documented cases of grade 4 liver injury [4]. Interestingly, the Japanese phase I and phase II trials (JPN-102, JPN-201, and JPN-202) for abiraterone acetate in CRPC showed a significantly higher incidence of liver injury. Liver injury affects approximately 80% of patients within three months of treatment initiation in Japan [9]. The incidence of DILI in Japanese patients was high, and the incidence of DILI in the group of upfront therapy in this study was significantly higher than in the LATITUDE trial group. Associations of human leukocyte antigen (HLA) genotypes and risk of DILI play an important role in the immune system in the pathogenesis of DILI [10]. Given the racial differences in HLA genotypes, we considered the possibility of racial differences in the onset of DILI. Therefore, these findings underscore the importance of monitoring liver function during the initial treatment phase. Thus, we recommend monitoring liver function at least every two weeks for the first three months and monthly monitoring thereafter.

A DILI can be categorized as hepatocellular, cholestatic, or mixed, based on the R ratio, which is the ratio of ALT to ALP relative to their respective upper limits of normal [7]. As defined by the Council for International Organizations of Medical Sciences (CIOMS) and modified by the Food and Drug Administration (FDA), an R ratio of > 5 indicates hepatocellular type DILI, < 2 indicates cholestatic type DILI, and 2-5 indicates mixed type DILI. The management of DILI primarily involves supportive care and close monitoring of clinical and laboratory parameters. Discontinuation of the offending medication is the first step because further progression of liver injury may lead to fulminant hepatitis and the requirement for liver transplantation [7]. In our study, all patients showed improvement following abiraterone acetate discontinuation, supportive management, and additional medications. These cases highlight the importance of pharmacological vigilance and close monitoring of liver function when administering abiraterone acetate to patients with prostate cancer.

Generally, it has been suggested that DILI is more likely to occur in females, the elderly, and patients with chronic liver disease, HIV, and obesity [11]. In our study, liver metastasis and abnormal γ-GTP levels were identified to be risk factors for DILI. Age was a very important factor, but it may not have been significant because many patients with prostate cancer were elderly. A high incidence of DILI in the chemotherapy-naive group of Japanese patients has been reported [9]; however, in this study, the incidence of DILI did not change with the treatment line.

Limitations

The present study has its limitations. First, this is a retrospective study, and the blood sampling intervals were not consistent. Second, a relatively small number of patients were enrolled in this study. Third, liver biopsy and aggressive imaging studies were not performed in all cases. Thus, there is a need for further studies to validate these findings and refine risk stratification.

## Conclusions

We investigated DILI caused by abiraterone acetate at Showa University Hospital. Severe DILI is a potential complication of abiraterone acetate administration. In our cases, it was found that DILI caused by abiraterone acetate improved relatively quickly by discontinuing the drug. The incidence of DILI in the group of upfront therapy in this study was significantly higher. A DILI develops within eight weeks. Therefore, liver function monitoring is recommended at least every two weeks for the first 12 weeks of treatment and every four weeks thereafter. Finally, clinicians must consider liver injury a complication during abiraterone acetate therapy, especially in the first few months.
